# Pretreatment patient-reported cognitive function in patients with diffuse glioma

**DOI:** 10.1007/s00701-022-05126-9

**Published:** 2022-02-10

**Authors:** Stine Schei, Ole Solheim, Øyvind Salvesen, Marianne Jensen Hjermstad, David Bouget, Lisa Millgård Sagberg

**Affiliations:** 1 Department of Public Health and Nursing, Norwegian University of Science and Technology, Trondheim, Norway; 2 Department of Neurology, St. Olavs hospital, Trondheim, Norway; 3 Department of Neuromedicine and Movement Science, Norwegian University of Science and Technology, Trondheim, Norway; 4 Department of Neurosurgery, St. Olavs hospital, Trondheim, Norway; 5 Unit for Applied Clinical Research, Department of Clinical and Molecular Medicine, Norwegian University of Science and Technology, Trondheim, Norway; 6 Regional Advisory Unit in Palliative Care, Department of Oncology, Oslo University Hospital, Oslo, Norway; 7 European Palliative Care Research Centre, Department of Oncology, Oslo University Hospital, Oslo, Norway; 8 Institute of Clinical Medicine, University of Oslo, Oslo, Norway; 9 Department of Health Research, SINTEF Digital, Trondheim, Norway

**Keywords:** Brain neoplasms, Cognition, Glioma, Patient-reported outcome measures, Preoperative period

## Abstract

**Purpose:**

Cognitive function is frequently assessed with objective neuropsychological tests, but patient-reported cognitive function is less explored. We aimed to investigate the preoperative prevalence of patient-reported cognitive impairment in patients with diffuse glioma compared to a matched reference group and explore associated factors.

**Methods:**

We included 237 patients with diffuse glioma and 474 age- and gender-matched controls from the general population. Patient-reported cognitive function was measured using the cognitive function subscale in the European Organisation for Research and Treatment of Cancer QLQ-C30 questionnaire. The transformed scale score (0–100) was dichotomized, with a score of ≤ 75 indicating clinically important patient-reported cognitive impairment. Factors associated with preoperative patient-reported cognitive impairment were explored in a multivariable regression analysis.

**Results:**

Cognitive impairment was reported by 49.8% of the diffuse glioma patients and by 23.4% in the age- and gender-matched reference group (*p* < 0.001). Patients with diffuse glioma had 3.2 times higher odds (95% CI 2.29, 4.58, *p* < 0.001) for patient-reported cognitive impairment compared to the matched reference group. In the multivariable analysis, large tumor volume, left tumor lateralization, and low Karnofsky Performance Status score were found to be independent predictors for preoperative patient-reported cognitive impairment.

**Conclusions:**

Our findings demonstrate that patient-reported cognitive impairment is a common symptom in patients with diffuse glioma pretreatment, especially in patients with large tumor volumes, left tumor lateralization, and low functional levels. Patient-reported cognitive function may provide important information about patients’ subjective cognitive health and disease status and may serve as a complement to or as a screening variable for subsequent objective testing.

## Introduction

Diffuse glioma is the most common primary malignant brain tumor in adults [36]. Cognitive impairment is a frequent symptom in glioma patients already before treatment [45]. This has a negative effect on patients’ and relatives’ quality of life [3, 38] and is independently associated with poor prognosis [28, 47]. That underlines the importance of detecting cognitive impairment and developing or tailoring treatments that preserve cognitive functions in this patient group. Unfortunately, cognitive difficulties are often underestimated and overlooked by clinicians [12].

Neuropsychological assessments with objective tests have been viewed as the gold standard of cognitive evaluation in glioma studies, but these may be lengthy, time-consuming, and burdensome for the patients, especially in unselected high-grade glioma patients. This may result in poor compliance and selection bias [4, 5, 17, 23]. Furthermore, extensive neurocognitive testing is primarily performed in small samples of selected patients with tumor location in specific brain areas and/or with specific symptoms [39, 45]. As a result, the generalizability of findings to the unselected brain tumor population may suffer. Importantly, objective tests do not necessarily reflect the patient’s subjective complaints. Therefore, in oncological glioma studies, the patient-reported cognitive function has been integrated to aid in establishing the net clinical benefit of oncological treatment [2, 21, 48], but assessments done before treatment are so far limited. Most available studies are also restricted to either cross-sectional design, lack of control groups, heterogeneous populations, or non-validated questionnaires [8, 10, 37, 43].

In the present study, we aimed to investigate the prevalence of preoperative patient-reported cognitive impairment in patients undergoing primary surgery for diffuse glioma compared to gender- and age-matched reference data from the Norwegian general population. Furthermore, we wanted to explore patient- and tumor-related factors that were associated with worse preoperative patient-reported cognitive function.

## Methods

### Study population

**Fig. 1:**
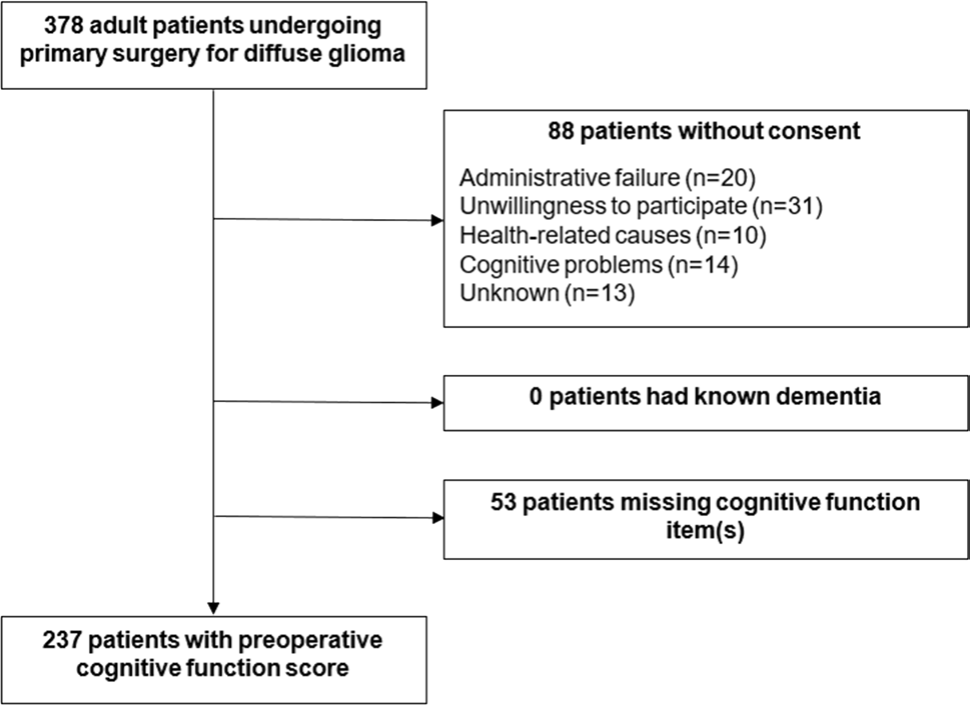
Flowchart showing the inclusion process

In this study, all patients with diffuse glioma aged ≥ 18 years scheduled for first time surgical resection or diagnostic biopsy at the Department of Neurosurgery, St. Olavs hospital, Trondheim University Hospital (Norway) from September 2011 to December 2019, were eligible for inclusion. This department serves a defined geographic catchment area with a population of approximately 750,000, ensuring population-based referral. All tumors were histopathologically verified as grade II–IV glioma according to the 2007 or 2016 World Health Organization classification [31, 32]. Exclusion criteria were known dementia. A total of 237 (63%) diffuse glioma patients were included in the analysis, and the inclusion process is presented in Fig. 1. The median age was 61 years (range 18–83 years), and 84 (35%) were females. Most patients had high-grade glioma (79%) and were functionally independent with a preoperative Karnofsky Performance Status (KPS) score ≥ 70 (86%). Only 5% had severe comorbidities (Table 1).

**Table 1:** Patient and tumor characteristics

	*N* = 237 (100)
Age in years, median (range)	61 (18–83)
Female, *n* (%)	84 (35)
Histopathology, *n* (%)	
Diffuse low-grade glioma	49 (21)
High-grade glioma	188 (79)
Tumor lateralization, *n* (%)	
Right	116 (49)
Left	116 (49)
Bilateral	5 (2)
Tumor location, *n* (%)	
Frontal	72 (30)
Temporal	44 (19)
Parietal	13 (6)
Occipital	2 (1)
Cerebellum/brainstem	2 (1)
Deep cerebral^a^	6 (2)
Multiple lobes	98 (41)
Preoperative Karnofsky Performance Status score ≥ 70, *n* (%)	203 (86)
Charlson Comorbidity Index ≥ 2, *n* (%)	12 (5)
Preoperative use of corticosteroids, *n* (%)	159 (67)
Preoperative use of antiepileptic drugs, *n* (%)	74 (31)
Preoperative tumor volume ml, median (range)^b^	26.77 (0.75–210.09)

^a^Basal ganglia/thalamus/corpus callosum/insula.

^b^*N* = 235 due to 2 missing MRI.

### Variables and data collection

All data were prospectively collected in a registry or as part of another project. Patient-reported cognitive function was measured using the cognitive function scale in the Norwegian-translated European Organisation for Research and Treatment of Cancer (EORTC) QLQ-C30 questionnaire (version 3) [1], which was completed 1–3 days before surgery by the patients themselves or with assistance from a nurse or family member. The cognitive function subscale includes two questions about concentration and memory. The time frame is the past week, and the questions are answered on a four-point scale from “not at all” to “very much.”

The KPS was rated prospectively by the operating neurosurgeon just before surgery [33]. In six patients, prospective KPS was missing and medical notes were used to retrospectively estimate if the patients were functionally dependent (< 70) or independent (≥ 70). Patient and treatment characteristics were collected from electronic medical records, and Charlson Comorbidity Index (CCI) was used to classify comorbidity [7]. Severe comorbidity was defined as CCI ≥ 2. Tumor volumes were estimated by semi-automatic tumor segmentation of MRIs using the software packages 3D Slicer version 4.3.1–4.10 (3D Slicer, Boston, Massachusetts) [14], or Brain Voyager™ QX version 1.2 (Brain Innovation, Maastricht, the Netherlands). The volume of pathological contrast-enhancement and necrotic tissue within the contrast-enhancing borders were used in contrast-enhancing tumors, while the entire volume as seen in T2/FLAIR sequences was used in tumors without ring contrast-enhancement. Lateralization was categorized according to where the center of mass in each tumor was located, while multifocal bilateral tumors were categorized as a separate group. Location was categorized based on which lobe that was involved. Tumors located in several lobes were categorized into a separate group.

### Reference population

An age- and gender-matched reference population was retrieved from previously collected representative sample data from the Norwegian population that was published in 1998 [26]. EORTC QLQ-C30 was sent out to 3000 Norwegian adults based on a random draw of all inhabitants. This population survey had a response rate of 66%, and 1926 persons answered the two questions regarding cognitive function. The reference population was matched with the glioma population on age and gender with the ratio of 2:1. The age- and gender-matched reference group counted 474 individuals, where the median age was 60 (range 19–83 years), and 35% were females.

### Statistical analyses

We used descriptive statistics to characterize the patients with diffuse glioma. Means are presented if data were normally distributed, while medians are presented if data were skewed.

The cognitive function questions were transformed into a 0–100 scale according to the EORTC scoring manual [13], with a cutoff of ≤ 75 indicating clinically important patient-reported cognitive impairment [20]. We examined dependence between cognitive function within matched triples using a mixed logistic regression model, with group as fixed effect and matched triple as random effect. Since the variance of the random effect was estimated at zero, the matched analysis of cognitive function simplified to an unmatched analysis. Therefore, Clopper–Pearson confidence interval (CI) for cognitive impairment proportion and Fisher’s exact test were used for comparing the diffuse glioma population with the reference population.

To investigate variables possibly associated with preoperative patient-reported cognitive impairment in patients with diffuse glioma, we used binary logistic regression analysis. Variables with a statistical trend (*p* < 0.1) in a univariable model were included in the final multivariable model. In all analyses, *p*-values ≤ 0.05 were considered statistically significant. The analyses were performed in R version 3.6.3 using the package lme4 (R Foundation for Statistical Computing, Vienna, Austria) and IBM SPSS Statistics for Windows, version 26 (IBM Corp., Armonk, N.Y., USA).

### Ethics

This study was approved by the Regional Committee for Medical and Health Research Ethics in South and East Norway (reference number 67005). All glioma patients provided written informed consent as part of other projects (reference number 2011/974 or 2015/215), and the data collection followed the Helsinki Declaration principles.

## Results

### Comparison between diffuse glioma patients and matched reference group

As shown in Table 2, 49.8% of the patients had a preoperative cognitive score ≤ 75, indicating a clinically important patient-reported cognitive impairment, compared to 23.4% in the matched reference group (*p* < 0.001). Thus, patients with diffuse glioma had 3.2 times higher odds for self-reported cognitive impairment compared to the matched reference group.

**Table 2:** Proportion (95% CI) of clinically important patient-reported cognitive impairment in diffuse glioma patients and the matched reference group

Diffuse glioma patients	Reference group	OR (95% CI)	*P*-value
118/237 49.8% (43.3, 56.3)	111/474 23.4% (19.7, 27.4)	3.24 (2.29, 4.58)	< 0.001*

*Indicates *p* ≤ 0.05.

*OR*, odds ratio; *CI*, confidence interval.

### Possible predictors of preoperative patient-reported cognitive impairment

In the univariable analyses, histopathology, tumor lateralization, KPS, use of corticosteroids, and tumor volume were factors significantly associated with patient-reported cognitive impairment (*p* ≤ 0.05), while there was a trend for gender (*p* < 0.1). When including these variables in a multivariable analysis, only tumor lateralization, KPS, and tumor volume remained significant independent predictors (Table 3). A tumor in the left hemisphere increased the odds for patient-reported cognitive impairment by 3.3 times compared to a tumor in the right hemisphere. For small tumors up to the 3rd quartile of volumes, there were increased odds for impairment with larger tumor volume, while increased KPS was associated with a slightly decreasing likelihood for reporting cognitive impairment. The concordance index, a measure of the predictive accuracy of the model, was 0.75.

**Table 3:** Factors associated with patient-reported cognitive impairment in diffuse glioma patients

Variables in the binary regression model	Univariable analyses		Multivariable analyses	
	OR (95% CI)	*p*-value	OR (95% CI)	*p-*value
Age	1.00 (0.99, 1.02)	0.680		
Female	1.59 (0.92, 2.70)	0.094*	1.83 (0.98, 3.41)	0.058
High-grade glioma	1.96 (1.02, 3.74)	0.042*	1.07 (0.43, 2.67)	0.890
Tumor lateralization		0.001*		< 0.001*
Right	Reference		Reference	
Left	2.68 (1.57, 4.55)	< 0.001*	3.30 (1.80, 6.06)	< 0.001*
Bilateral	1.09 (0.17, 6.79)	0.926	0.54 (0.08, 3.56)	0.519
Tumor location		0.478		
Frontal	Reference			
Temporal	1.35 (0.64, 2.88)	0.432		
Parietal	2.37 (0.71, 7.98)	0.163		
Occipital	-	-		
Cerebellum/brainstem	1.48 (0.09, 24.67)	0.784		
Deep central	0.74 (0.13, 4.32)	0.739		
Multiple lobes	1.90 (1.02, 3.52)	0.042*		
Karnofsky Performance Status (continuous)^a^	0.96 (0.94, 0.98)	< 0.001*	0.96 (0.94, 0.99)	0.006*
Charlson Comorbidity Index ≥ 2	0.71 (0.22, 0.30)	0.565		
Corticosteroids	1.99 (1.14, 3.45)	0.015*	1.04 (0.46, 2.38)	0.918
Antiepileptic drugs	0.74 (0.42, 1.28)	0.282		
Tumor volume^b^		0.002*		0.028*
≤ 9.7 ml	Reference		Reference	
9.8–26.8 ml	1.78 (0.84, 3.76)	0.133	1.76 (0.77, 4.05)	0.179
28.5–56.5 ml	4.43 (2.04, 9.60)	< 0.001*	3.93 (1.60, 9.69)	0.003*
56.9–210.1 ml	2.42 (1.14, 5.12)	0.021*	1.88 (0.74, 4.75)	0.184

*Indicates *p* ≤ 0.05.

*OR*, odds ratio; *CI*, confidence interval.

^a^*N* = 231 due to 6 missing KPS score.

^b^Quartiles. *N* = 235 due to 2 missing MRI.

For KPS, there was a relationship where lower functional levels were associated with higher frequencies of patient-reported cognitive impairment. Even in patients with normal functional levels (KPS 100), 37% of the patients reported cognitive impairment (Table 4). The risk of patient-reported cognitive impairment was significantly higher in a subgroup of patients with normal or close to normal functions (KPS 90–100) compared to matched references (38% vs. 21%, OR 2.27, 95% CI 1.33, 3.78, *p* = 0.002).

**Table 4:** Frequency of patient-reported cognitive impairment across different functional levels

Karnofsky Performance Status score	Clinically important cognitive impairment	
	Yes (*N* = 115)^a^ *n* (%)	No (*N* = 116)^b^ *n* (%)
100	13 (37)	22 (63)
90	29 (39)	46 (61)
80	22 (48)	24 (52)
70	29 (67)	14 (33)
60	13 (62)	8 (38)
50	9 (82)	2 (18)

^a^*N* = 115 due to 3 missing KPS. ^b^ *N *= 116 due to 3 missing KPS.

## Discussion

In this study, we assessed pretreatment patient-reported cognitive impairment in patients with diffuse glioma. Almost half of the patients reported a clinically important cognitive impairment, which was more than two times as prevalent as in the matched reference group from the general population. Patient-reported cognitive impairment was also common in patients with normal or close to normal functional levels. Independent predictors for preoperative patient-reported cognitive impairment were tumors located in the left hemisphere, large tumor volume, and lower functional levels. These findings indicate a potential validity of patient-reported cognitive function in glioma patients.

The high prevalence of patient-reported cognitive impairment in our study is in line with findings in a systematic review of studies using objective neurocognitive tests that found cognitive impairment in approximately 60% of glioma patients before surgical treatment [45]. Even if there has been an increasing emphasis on incorporating patient-reported data in neurosurgical clinical research in the last years, studies examining patient-reported cognitive function are still rare. In a cross-sectional study using the EORTC questionnaire in glioma patients before primary and recurrent surgery [8], cognitive impairment had a prevalence of 75%. However, here, they had merged the social and cognitive function scales and did not report cutoff scores when defining cognitive impairment. Also, unstandardized assessments have been used in a small study of low-grade glioma patients where 33% reported preoperative problems with concentration the last year [10], and in a larger brain tumor study where 32% and 24% reported problems with memory and attention before treatment, respectively [43].

The high prevalence of patient-reported impairment in the glioma population is probably caused by a variety of factors. In neuropsychological studies, there is strong evidence that the tumor itself causes impairment [30], which in the present study also seems to apply to patient-reported impairment. Corticosteroids and antiepileptic drugs are other factors known to potentially contribute to declined objective cognitive function [23, 29], although we did not find any such associations with patient-reported function. Similarly, no association between patient-reported cognitive function and antiepileptic drugs was found in another study [44]. Additionally, the psychological effect of being diagnosed with a life-threatening disease and additional symptoms such as fatigue and sleep disturbance may hamper the patient’s ability to concentrate and remember things [16, 18, 37, 40].

We found an increased risk of patient-reported cognitive impairment with increasing tumor volume up to the third quartile of volumes. A relationship between large tumor volume and/or mass effect and cognitive impairment is found in previous studies using objective tests as well [19, 23, 25, 42, 46]. Furthermore, better patient-reported cognitive functioning, as measured with EORTC cognitive function scale, is found to be independently associated with both overall survival and progression-free survival in glioma patients [11]. Thus, our results support what others have suggested, that patients’ cognitive complaints may reflect tumor burden and may therefore be an indicator of the severity of the disease [11]. Interestingly, in our material, the risk of patient-reported cognitive impairment was somewhat lower in patients harboring the largest quartile of tumor volumes. Since few patients had very large tumor volume, this finding may be due to chance. Large tumors may also cause other symptoms overshadowing cognitive problems, such as nausea/vomiting, aphasia, and motor deficits, and are also more often located in the frontal lobe and may affect the patient’s self-awareness [9].

Our findings suggest that tumors in the left hemisphere negatively affect patients’ self-perceived cognitive function, and also in the literature, left-sided tumors appear to cause more severe objective cognitive deficits compared to right-sided tumors [23–25, 34]. However, damage in the right hemispheric is found to be associated with anosognosia [35, 41]. Thus, it is reasonable to assume that patients with right-sided tumors report less subjective cognitive impairment due to impaired awareness. However, a recent study found no difference between self-awareness in HGG patients with left-sided and right-sided tumor lateralization [22]. Furthermore, an association between affected lobes and worse performance on specific objective tests has been reported [19, 25]. Few patients in each lobe may explain why we did not find such association. In addition, since the EORTC questionnaire only contains two questions regarding memory and concentration, we acknowledge that this scale is not sensitive enough to detect all mental abilities or to provide a thorough assessment of patient-reported cognitive function. On the other hand, it gives an indication of a common patient problem that warrants further examination.

In our material, patient-reported cognitive impairment was common also in patients who, according to the surgeons, had more or less normal functional levels. This is important knowledge since subtle cognitive symptoms may have a large impact on patients’ quality of life and ability to work. We also found that lower functional levels increased the risk of subjective cognitive impairment, which is in accordance with previous findings [8]. Our finding is not surprising since KPS does not distinguish among different types of performance limitations [28]. In addition, cognitive function and KPS share the same risk factors, such as higher age, high-grade histology, and larger tumor volume.

Patient-reported outcomes have not been validated to measure cognitive function in brain tumor patients, and there may be a low correlation between patient-reported data and objective neurocognitive assessment [6, 18]. Thus, it is suggested not to use patient-reported outcomes as a surrogate of objective neuropsychological functioning [6]. Yet, this should not diminish the importance or relevance of the patient’s subjective complaints, and several recommend using a combination of objective and patient-reported outcome measures of cognitive functioning [18, 27]. The clinical experience is that some patients experience a discrepancy between their present and previous cognitive function, even if the results from the neuropsychological tests are normal. Patient-reported questionnaires can be a practical tool to provide information about patients’ cognitive health, especially in unselected patients who are not suitable for extensive neuropsychological testing.

The main strength of this study is the large prospective population-based data collection and the matched control group, increasing the generalizability of our findings. The study also has some limitations. First, we attempted to include unselected glioma patients, but cannot exclude an extent of selection bias and a potential underestimation of patient-reported cognitive impairment. Second, we did not have information about the patients’ level of education or material status, which may be important factors for patient-reported cognitive function. Furthermore, the reference material was published in 1998, but a national population survey was repeated in 2004 with similar scores on the EORTC cognitive function scale [15]. Additionally, even though the patients’ self-awareness may perhaps be questionable, we argue that ratings from their perspective should not be ignored due to the significant impact on their quality of life. Moreover, two questions from the EORTC questionnaire do not capture all aspects of how patients experience their cognitive function, and more subtle impairments could probably be detected with a more detailed questionnaire. However, such a questionnaire may have been too complicated for our unselected glioma patients. At last, since some patients received assistance when scoring the questionnaire, we cannot exclude that the answers were influenced by others.

## Conclusion

We investigated pretreatment patient-reported cognitive impairment in patients with diffuse glioma. We found that almost half of the diffuse glioma patients reported cognitive impairment before surgery, which was almost twice as much as in the general population. It was a common symptom also in patients with normal surgeon-reported functional levels. Independent predictors were left-sided tumors, large tumor volume, and lower functional levels. Patient-reported cognitive function may provide important information about unselected glioma patients’ subjective cognitive health and disease status, and serve as a complement or a screening variable for subsequent objective testing.

## Data Availability

The dataset generated during and/or analyzed during the current study is not publicly available due to privacy concerns but is available from the corresponding author on reasonable request.

## Abbreviations

EORTC: European Organisation for Research and Treatment of Cancer
KPS: Karnofsky Performance Status
CCI: Charlson comorbidity index
MRI: Magnetic resonance imaging
3D: Three-dimensional
FLAIR: Fluid-attenuated inversion recovery
CI: Confidence interval
OR: Odds ratio
